# The Opposite Functions of CD30 Ligand Isoforms

**DOI:** 10.3390/cimb46030172

**Published:** 2024-03-21

**Authors:** Ignat Printsev, Elyas Alalli, Janine Bilsborough

**Affiliations:** F. Widjaja Foundation Inflammatory Bowel and Immunobiology Research Institute, Cedars-Sinai Medical Center, Los Angeles, CA 90048, USA; elyas.alalli@cshs.org (E.A.); janine.bilsborough@cshs.org (J.B.)

**Keywords:** CD30, CD30 ligand, inflammatory bowel disease, protein isoforms, cell signaling

## Abstract

TNFSF8/CD30 ligand is a TNF superfamily member expressed on several major immune cell types, including activated monocytes, B, and T cells. The signaling of CD30 ligand through its cognate CD30 receptor has been shown to have effects on cell differentiation, cell death/survival, and cytokine production. The signaling pair has been implicated in hematopoietic malignancies and inflammatory disease, and a chemotherapy–CD30 antibody combination for the treatment of Hodgkin and other lymphomas has been developed. There are two recorded isoforms of CD30 ligand. All hitherto studies of CD30 ligand are of the first, canonical isoform, while the second isoform has never been described. This study aims to elucidate the properties and signaling functions of the second CD30 ligand isoform. We have found mRNA expression of both isoforms in the PBMCs of all six healthy donors tested. Through methods in cell biology and biochemistry, we were able to discover that the second CD30 ligand isoform has no discernable pro-inflammatory function and, in fact, isoform 2 can restrict the capacity of the canonical isoform to signal through the CD30 receptor by preventing their interaction. This discovery has implications for the future development of therapeutics targeting the CD30/CD30 ligand signaling pair in cancer and inflammatory disease.

## 1. Introduction

TNFSF8 encodes the protein CD30 ligand (CD30L), a TNF superfamily member and a single-pass type II membrane protein with its C-terminus in the extracellular space. CD30L is the cognate ligand for the CD30 receptor, encoded by TNFRSF8. The receptor was originally discovered as a specific marker for Hodgkin’s lymphoma and subsequently other hematopoietic malignancies, with its expression normally limited to activated T and B lymphocytes [1,2]. CD30L is expressed on activated monocytes, B, and T cells, and the CD30/CD30L signaling pair has been shown to have pleiotropic downstream effects including differentiation, cell survival and death, NFkB activation, and production of cytokines [3,4,5]. Due to the presence of CD30 on the surface of cancer cells, there has been considerable interest in the receptor as a target for anti-tumor biologics and cancer immunotherapy, leading to the development of targeted treatments such as the chemotherapy–CD30 antibody combination brentuximab vedotin (BV) for the treatment of Hodgkin’s and other lymphomas [6,7,8].

Not surprisingly, given their role in cellular pathways related to inflammation, the CD30/CD30L signaling pair has been implicated in immune diseases such as rheumatoid arthritis [9], allergic airway inflammation [10], and inflammatory bowel disease (IBD). A soluble form of the CD30 protein (sCD30) has been documented at higher levels in the sera of patients with both ulcerative colitis (UC) and Crohn’s disease (CD), compared to healthy controls, although some studies point to a higher prevalence of sCD30 in UC over CD [11,12]. CD30 expression on eosinophils in the colon was found to be a highly effective differentiating marker of UC versus CD in IBD patients [13], and studies have suggested that circulating monocytes from UC patients have elevated levels of CD30L [14,15]. More recently, genetic association studies in humans have identified single nucleotide polymorphisms (SNPs) within the TNFSF8 locus associated with risk of IBD [16,17,18]. In in vivo mouse colitis models, CD30L is involved in mediating inflammation in the gut. Genetic deletion of CD30L or treatment with anti-CD30 antibodies in vivo results in resistance to DSS-induced colitis, including attenuated cytokine production and preservation of colon length and body weight [19,20]. Together, these data suggest that CD30L may be a therapeutic target for IBD.

In humans and primates, two transcript variants for CD30L have been reported. CD30L isoform 1 (Iso1) is the longer isoform and the subject of all prior studies concerning CD30L function. CD30L isoform 2 (Iso2) has never been characterized. Therefore, in this study, we have investigated the function of CD30L isoform 2 and its impact on CD30-mediated inflammatory signaling.

## 2. Materials and Methods

### 2.1. Ethics Statement 

Six healthy adult blood samples were collected in accordance with the Declaration of Helsinki and approved by the Institutional Review Board of Cedars-Sinai Medical Center (IRB 3358, continuously reapproved for this project work since 19 August 2019). All subjects gave their informed consent in writing prior to their inclusion in this study.

### 2.2. Sequences and Structural Homology Prediction 

CD30L isoform sequences were gathered from the National Center for Biotechnology Information (NCBI) gene database, accession numbers NP_001235.1 and NP_001239219.1. Putative protein domain and topology were gathered from UniProt, accession P32971. Sequence alignment was performed with Clustal Omega [21]. The Phyre2 web portal V2.0 [22] was used to generate secondary structure models of the extracellular domains of the CD30L isoforms using the intensive setting. 

### 2.3. PBMC Isolation and Stimulation

Blood was drawn in sodium heparin tubes from six healthy donors. Peripheral blood mononuclear cells (PBMCs) were isolated by gradient centrifugation with Lymphocyte Separation Medium (Corning 25-072-CV, Corning, NY, USA) according to the manufacturer’s recommended protocol. PBMCs were plated and stimulated in PBMC media: RPMI with Glutamax (ThermoFisher 72-400-120, Waltham, MA, USA) containing 10% fetal bovine serum (Omega Scientific FB-02, Tarzana, CA, USA) and 1% penicillin–streptomycin solution. PBMCs were cultured and stimulated at 1 × 10^6^ cells/mL. Stimulations were performed for the time lengths indicated in each experiment.

Immune complex (IC) stimulation involved coating treated tissue culture plates with 0.5 mg/mL human IgG (Jackson ImmunoResearch Labs 009-000-002, West Grove, PA, USA) in PBS for 30 min at room temperature or in a humidified container overnight at 4 °C. Wells were then washed with PBS and coated with 20 µg/mL mouse anti-human IgG (JIR 209-005-098) in PBS for 1 h. Wells were washed again before the addition of PBMCs. Stimulation by using Dynabeads with anti-CD3 and anti-CD28 antibodies (ThermoFisher 11131D, Waltham, MA, USA) was performed based on the manufacturer’s recommendations. Briefly, 25 µL of Dynabeads per 2 × 10^6^ PBMCs were used. Beads were washed with PBS, resuspended in PBMC media, and added to cells. Stimulation with PMA (Sigma-Aldrich P1585, Saint Louis, MO, USA) and ionomycin (Sigma-Aldrich I3909, Saint Louis, MO, USA), demarcated as P/I in the figures, was performed at 2.5 ng/mL PMA and 0.5 µM ionomycin. Since both PMA and ionomycin are in a DMSO solution, DMSO at equal *v*/*v* concentrations was used as a vehicle control condition. Stimulation with LPS (Millipore LPS25, Burlington, MA, USA) was conducted at a final concentration of 1 µg/mL.

### 2.4. CD30L Isoform qPCR

For RNA isolation, 2 × 10^6^ PBMCs were used. Cells were spun down in their culture wells after stimulation, the supernatant was aspirated, and PBMCs were processed using QIAShredder tubes and the Qiagen RNeasy kit (Qiagen 79656 and 74104, Germantown, MD, USA). If the samples were to be processed later, the lysates in Buffer RLT were frozen at −80 °C. cDNA was synthesized using the ProtoScript^®^ II kit (New England BioLabs M0368X, Ipswich, MA, USA). qPCR was performed by using the SsoAdvanced™ Universal SYBR^®^ Green Supermix (Bio-Rad 1725271, Hercules, CA, USA) on a Bio-Rad CFX Opus Real-Time PCR System using the following primers: Actin-F, GATGACCCAGATCATGTTTGAGACCTTCAACACC; Actin-R, CGCGCTCGGTGAGGATCTTCATGAGGTAG; Iso1-F, CCTACCTCCAAGTGGCAAAG; Iso1-R, CTTCAGATCGACAGAATTATTTGGG; Iso2-F, CCAATTCCCTGATTACTGTGGC; Iso2-RGGGTTGTAGAGTTTCAAGGCA. Beta actin (NM_001101.5) was used as a normalization control and data were analyzed using Bio-Rad CFX Maestro software v5.3.022.1030. The primers yielded single bands on an agarose gel and single peaks during melting curve analysis.

### 2.5. Cell Culture, Viral Transduction, and Clonal Isolation

Flp-In™ 293 T-REx, referred to as HEK in this text, were from ThermoFisher (R78007, Waltham, MA, USA) and were cultured in DMEM with 10% fetal bovine serum (Omega Scientific FB-02) and 1% penicillin–streptomycin. KARPAS 299 (K299) were a gift from Prometheus Biosciences (San Diego, CA, USA). K299 and its derivatives were cultured in RPMI with Glutamax (ThermoFisher 72-400-120, Waltham, MA, USA) containing 20% fetal bovine serum and 1% penicillin-streptomycin. KM-H2 and HDLM-2 were from the German Collection of Microorganisms and Cell Cultures (DSMZ ACC8 and DSMZ ACC17, Braunschweig, Germany) and cultured in RPMI with Glutamax (ThermoFisher 61870036, Waltham, MA, USA) containing 20% fetal bovine serum and 1% penicillin–streptomycin. Cells were cultured at 37 °C under 5% CO_2_.

K299 cells were transduced with Cignal Lenti NFkB Reporter (Qiagen CLS-013L-1, Germantown, MD, USA) lentivirus particles at 16× MOI in 96-well plates at 800K cells/mL. Transduction was performed by using hexadimethrine bromide (polybrene) (SigmaAldrich H9268-5G, Saint Louis, MO, USA) at 8 µg/mL final concentration and centrifugation for 2 h at 1000 RCF at 32 °C. Then, 24 h after transduction, the media was changed, and cells were allowed to grow for 48 more hours before the addition of puromycin at 1.5 µg/mL for reporter selection. After 7–10 days, the population was considered pure and was referred to as K299-NFkB-Luc pooled clones. For clonal selection of K299-NFkB-Luc, cells were single-cell sorted into 96-well plates containing 30% conditioned media from growing K299 cells. The BD FACSAria™ III Sorter was used to single-cell sort. Clones were tested and chosen for response to CD30L-Fc recombinant protein (Sino Biological 10040-H01H, Wayne, PA, USA) via luciferase assay (described below).

### 2.6. Flow Cytometry

Flow cytometry of HEK cells and PBMCs was conducted under non-permeabilizing conditions. Cells were harvested into PBS or Versene (ThermoFisher 15040-066, Waltham, MA, USA), washed, and then stained with live/dead stain (ThermoFisher L34964, Waltham, MA, USA) for 30 min in PBS per the manufacturer’s recommendations. Cells were washed again and resuspended in eBioscience™ Flow Cytometry Staining Buffer (ThermoFisher 00-4222-26, Waltham, MA, USA). PBMCs were additionally treated with Human TruStain FcX™ Fc Receptor Blocking Solution (BioLegend 422302, San Diego, CA, USA) at 5% final *v*/*v* for 15 min. Labeled antibodies were used for staining with dilutions of 1:100 as follows: anti-human CD30 ligand (R&D FAB1028G, FAB1028S, Minneapolis, MN, USA), anti-FLAG M2-FITC (Sigma-Aldrich F4049, Saint Louis, MO, USA), and the following antibodies from BD Biosciences (Franklin Lakes, NJ, USA): anti-human CD30 (550041), anti-human CD14 (B555397); anti-human CD19 (555413); anti-human CD3 (555342).When multiple fluorescent labels were used in one assay, compensation was performed by the manufacturer’s recommendation using the AbC Total Antibody Compensation Bead Kit (ThermoFisher A10497, Waltham, MA, USA) and ArC Amine Reactive Compensation Bead Kit (ThermoFisher A10346, Waltham, MA, USA). Samples were processed using the Attune NxT Flow Cytometer and AutoSampler (ThermoFisher, Waltham, MA, USA).

### 2.7. Expression of CD30L Isoforms

Open reading frames (ORFs) for both the CD30L isoforms were purchased from Origene (RC211276, RC231923, Rockville, MD, USA), containing C-terminal FLAG/MYC epitope tags. ORFs were subcloned into the pcDNA5/FRT/TO (ThermoFisher V652020, Waltham, MA, USA) vector with or without epitope tags (e.g., wild type sequence Iso1, 1NT, or FLAG-tagged sequence Iso1-FL, 1FL). Vectors were transfected into Flp-In™ 293 T-REx cells (ThermoFisher R78007, Waltham, MA, USA), referred to as HEK cells in this text, using the polyethylenimine method [23] and allowed to express for 48 h. Transfections with multiple plasmids were carried out with plasmids in equal amounts, except where noted otherwise. Flp-In™ 293 T-REx (HEK) cells constitutively express the Tet repressor and Tet operator-containing plasmids such as pcDNA5/FRT/TO require the presence of doxycycline for expression, so 1 µg/mL doxycycline was used for expression induction. Transfections were performed in full HEK media, DMEM with 10% fetal bovine serum, and 1% penicillin–streptomycin solution, or in serum- and antibiotic-free media if the supernatants were to be used in a plate-based binding assay.

### 2.8. Plate-Based Binding and Cytokine Assays

Lysates or supernatants from transfected HEK cells were used for plate-based binding assays with the Meso Scale Diagnostics (MSD) system. MSD 96 well plates (MSD L15XA-3, Rockville, MD, USA) were coated overnight at 4 °C with soluble recombinant CD30 protein with or without Fc fusion (R&D 813-CD-100 or 6126-CD-100, Minneapolis, MN, USA), as noted. Coating antibodies included Iso1-specific anti-CD30L (R&D MAB1028, Minneapolis, MN, USA), pan-anti-CD30L (LSBio C293348, Shirley, MA, USA), and anti-FLAG (Sigma-Aldrich F3165, Saint Louis, MO, USA). Coating was performed at 1 µg/mL in PBS. Wells were washed with PBS with 0.1% Tween-20 (PBST) three times between every step. After coating, wells were blocked with Stabilcoat (Sigma-Aldrich S0950-1L, Saint Louis, MO, USA). Samples were either clarified lysates or supernatants (from serum free media) from HEK cells transfected with CD30L isoforms. Lysates were made with lysis buffer (1% Triton, 20 mM Tris pH 7.4, 100 mM NaCl, 1 mM MgCl_2_, 10% glycerol) + protease inhibitors (ThermoFisher 78442, Waltham, MA, USA) and clarified by centrifugation at 16K RCF for 5 min. Lysates and supernatants were added to the wells undiluted. Detection primary antibodies were diluted to 1:200 in PBST with 1% BSA and included rabbit anti-FLAG (CST 14793S, Danvers, MA, USA), and mouse Iso1-specific anti-CD30L (R&D MAB1028, Minneapolis, MN, USA). Secondary antibodies were diluted to 1:1000 in PBST with 1% BSA and included goat anti-rabbit SULFO tag (MSD R32AB, Rockville, MD, USA), and goat anti-mouse SULFO tag (MSD R32AC, Rockville, MD, USA). Plates were read by electrochemiluminescence (ECL counts) with 2× MSD Read Buffer (MSD R92TC, Rockville, MD, USA) diluted in water from 4× by using the MESO QuickPlex SQ 120MM instrument (MSD, Rockville, MD, USA). Cytokines were measured from tissue culture supernatant by using the MSD V-PLEX Pro-inflammatory Panel 1 Human Kit (MSD K15049D, Rockville, MD, USA), according to the manufacturer’s recommendations.

### 2.9. CD30L Isoform Stimulation and Reporter Assays

Recombinant Iso1 CD30L-Fc protein (Sino Biological 10040-H01H, Wayne, PA, USA) and transfected HEK cell culture were used to stimulate CD30+ cells. Co-culture stimulation involved seeding HEK cells at 4000 cells per well in Corning^®^ BioCoat™ Poly-D-Lysine 96-well microplates (Corning 354461, Corning, NY, USA) to assist with adherence during media changes. Cells were usually seeded in 3–8 replicates to minimize readout variation. HEK cell titration experiments started from 6000 cells per well and were serially diluted to 1:1 down to 47 cells before seeding. Seeded HEK cells were allowed to adhere overnight and transfected for 48 h in DMEM-based media, after which CD30+ cells (either wild type or reporter cells) were added at 50K cells in 150 µL fresh RPMI-based media per well. CD30+ cells were stimulated for 24 h in co-culture before supernatant harvest for cytokine analysis or luciferase assay for NFkB reporter activation. Luciferase assays were carried out using the Luciferase Assay System (Promega E1501, San Luis Obispo, CA, USA) based on the manufacturer’s recommendations. Briefly, cells were spun down, supernatants were removed, and cells were washed once in PBS. Then, 20 µL of lysis buffer (Promega E3971, San Luis Obispo, CA, USA) was added per well and the plates were frozen and thawed before 15 µL lysate was used in the assay with 100 µL of assay reagent. Luminescence was read by using the BioTek Cytation 5 plate reader (BioTek, Winooski, VT, USA).

### 2.10. Co-Immunoprecipitation and Western Blotting

HEK cells were seeded in 10 cm plates at 3 × 10^6^ cells per plate overnight and transfected with 0, 625, or 2500 ng Iso1 (1NT) plasmid with or without 2500 ng Iso2-FL (2FL) plasmid. Each transfection had a total of 5000 ng plasmid DNA with the addition of empty vector plasmid if necessary. CD30L isoforms were expressed for 48 h with 1 µg/mL doxycycline, lysed in lysis buffer with inhibitors (see above recipe), and clarified by centrifugation at 16K RCF for 5 min. Then, 10% of the lysate was saved as the input fraction whole-cell lysate, and the remainder was mixed with 5 µg of CD30-Fc recombinant protein (R&D 813-CD-100) and incubated for 1 h. 30 µL of protein A/G beads (Santa Cruz sc-2003, Santa Cruz, CA, USA) were added to the mixtures and incubated for an additional hour. Beads were washed 8 times in lysis buffer without protease inhibitors and on the final wash, the supernatant was completely removed. Following this, 2× SDS-PAGE buffer with 100 mM DTT was added at equal volumes to the input lysates and bead pellet and boiled for 5 min at 95 °C before being loaded onto 4–12% gradient Bis–Tris gels. Gels were transferred to nitrocellulose using the iBlot system (ThermoFisher, Waltham, MA, USA). Membranes were blocked with 5% milk in tris buffered saline with 0.1% Tween-20 (TBST) and incubated with the following primary antibodies in 2.5% milk TBS: anti-pan-CD30L (R&D BAF1028, Minneapolis, MN, USA), anti-CD30 (Abcam ab23766, Cambridge, MA, USA), anti-FLAG (Sigma-Aldrich F3165, Saint Louis, MO, USA), anti-GAPDH (Santa Cruz sc-32233, Santa Cruz, CA, USA). Secondary antibodies included: anti-mouse IgG HRP-linked (CST 7076S, Danvers, MA, USA), anti-biotin HRP-linked (CST 7075S, Danvers, MA, USA). Membranes were developed by using SuperSignal™ West Pico PLUS Chemiluminescent Substrate (ThermoFisher 34577, Waltham, MA, USA) and SuperSignal™ West Atto Ultimate Sensitivity Substrate (ThermoFisher A38554, Waltham, MA, USA) using the AlphaImager Gel Imaging System (Alpha Innotech, San Leandro, CA, USA). Densitometry was performed using standard methods by using ImageJ v1.53n (NIH, Bethesda, MD, USA).

### 2.11. Graphs and Statistical Analyses

The error bars in the bar graphs and XY plots indicate standard deviations from the mean. *p* values were generated using a two-tailed, unpaired *t*-test. Graphing and statistical analyses were carried out using GraphPad Prism v9.2.0 (GraphPad, Boston, MA, USA).

## 3. Results

### 3.1. CD30L Has Two Isoforms

**Figure 1:**
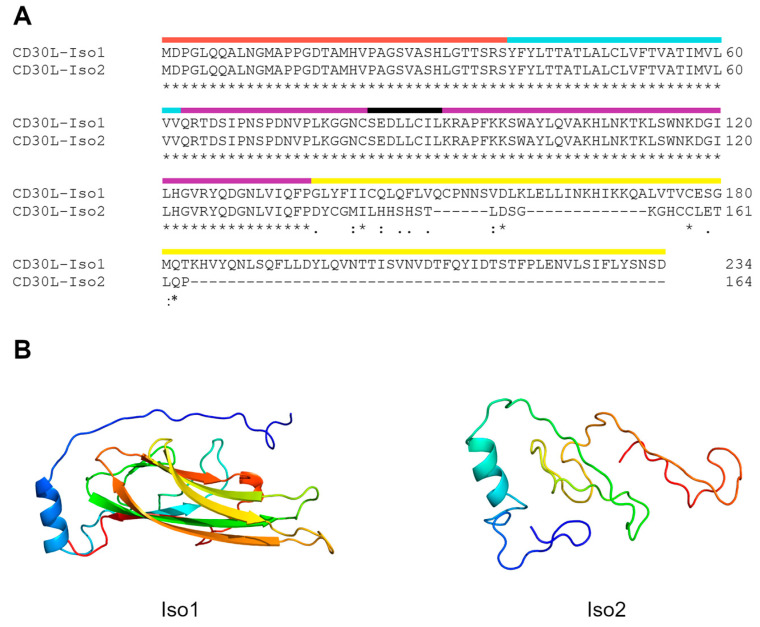
The CD30 ligand isoforms have distinct C-terminal extracellular regions. (**A**) Protein alignment of the two isoforms using Clustal Omega. Red—shared intracellular domain; cyan—shared transmembrane domain; purple—shared extracellular domain; black—predicted alpha helix within shared extracellular domain; yellow—extracellular sequences unique to each isoform. Asterisks indicate matching amino acid identity, dots and double dots indicate similarity, and hyphen indicates absence of residues in Iso2. The N-terminus for both proteins is intracellular and identical through residue 136, which includes the transmembrane domain at residues 38–62. The residues following 136 are distinct extracellular C-termini. (**B**) The predicted structural homology of CD30L isoforms to known protein structures generated by the Phyre2 web portal. Iso1 (**left**) showed strong homology to human TNF-alpha with several antiparallel beta-sheets. However, the software did not predict the presence of strong secondary structures for Iso2 (**right**), instead matching homology to small portions of proteins with unstructured domains. The two isoforms did share a predicted alpha helix that was membrane proximal on the extracellular side, present on the left side of each image.

CD30L isoform 2 (Iso2) is the product of an alternative splice site in the coding region of the last exon, and the result of this is a shorter protein that is partially distinct from Iso1 in its C-terminal extracellular domain (Figure 1A). Structural predictions based on the sequences of the extracellular domain with Phyre2 V2.0 software have found homology between Iso1 and human tumor necrosis factor alpha (TNF-alpha, PDB 4TSV) with a series of antiparallel beta-sheets. However, in an assessment of Iso2, there were no homologous proteins that contained a clearly defined secondary structure, with results including several short sequences from TNF-family members (PBD 4MSV, 4TSV) which were almost entirely disordered and varied greatly between candidate homologs. The main exception to this lack of secondary structure and homology between the CD30L isoforms was a predicted alpha helix in a completely homologous membrane-proximal region (Figure 1B).

### 3.2. CD30L Isoforms Are Expressed in Primary Cells

**Figure 2:**
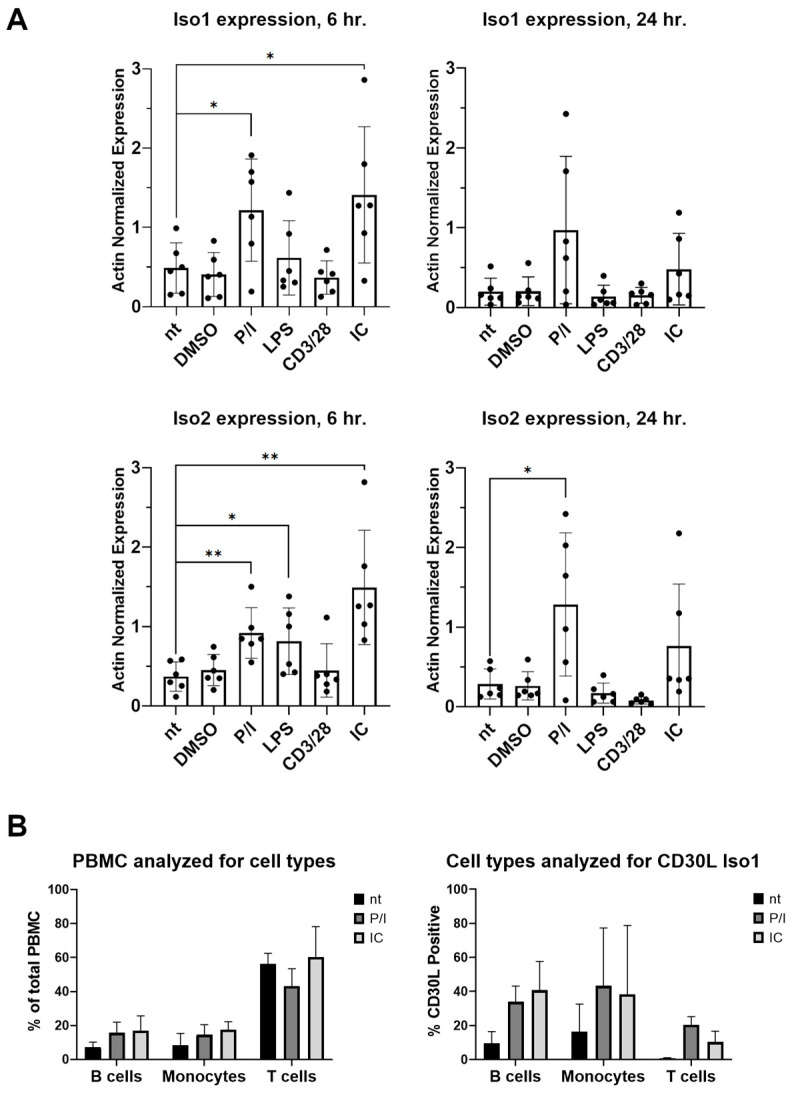
Both CD30L isoforms were expressed at the RNA level in primary human cells. (**A**) PBMCs were isolated from healthy donors (*n* = 6). Cells were either untreated (nt) or stimulated with DMSO, PMA/ionomycin (P/I), lipopolysaccharide (LPS) isolated from *E*. *coli* 0111:B4 (LPS), anti-CD3/CD28 beads (CD3/28), or immune complex (IC) for 6 or 24 h. The graphs show actin-normalized expression of both isoforms at both timepoints for all stimulation conditions. Single asterisks indicate *p* ≤ 0.05 and double asterisks indicate *p* ≤ 0.01 between the conditions indicated. Error bars represent standard deviation between donors. (**B**) PBMCs were isolated from healthy donors (*n* = 3, half of the 6 total donors). Cells were either untreated (nt) or stimulated with PMA/ionomycin (P/I) or immune complex (IC) for 24 h. After incubation, cells were subject to flow cytometry for B cell marker CD19, monocyte marker CD14, and T cell marker CD3, as well as CD30 ligand Iso1. PBMC cell types were present at expected ratios. Error bars are standard deviation between donors.

To confirm the presence of both CD30L isoforms in primary human cells, qPCR was performed on peripheral blood mononuclear cells (PBMCs) isolated from six healthy donors. PBMCs were stimulated with immune complex (IC), PMA/ionomycin (P/I), anti-CD3/CD28 beads, and lipopolysaccharide (LPS). At 6 h of stimulation, expression patterns for the isoforms were similar, with elevated transcripts in PMA/ionomycin, LPS, and IC conditions compared to untreated (nt) cells (Figure 2A). At 24 h of stimulation, isoforms were no longer expressed above control following stimulation with LPS or IC, with only PMA inducing both transcripts. We were only able to detect endogenous Iso1 at the protein level, as the commercially available antibodies for CD30L do not detect Iso2 by flow cytometry (Figure S1). In stimulated PBMCs from three out of six healthy donors, Iso1 protein was detected in monocytes, B cells, and stimulated T cells. T cells make up the largest percent of the PBMC population, and therefore are likely the largest contributor of CD30L in PBMCs, despite only 10–20% of T cells expressing the CD30L Iso1 protein after stimulation. However, monocytes express the highest percent by population, with an average of around 40% of monocytes expressing CD30L Iso1 after stimulation (Figure 2B).

### 3.3. CD30L Isoforms Are Present in a CD30 Protein Complex

Due to the differences in the extracellular C-terminal regions between the CD30L isoforms, we hypothesized that they would have some significant differences in their signaling and interaction properties, especially with regard to binding CD30. Because of difficulties in creating high-quality recombinant proteins of both CD30L isoforms, we relied on expressing CD30L isoforms in T-REx HEK (HEK) cells and the lysates derived from those cells. HEK cells do not endogenously express either CD30L isoform as measured by semi-quantitative RT-PCR or CD30 as measured by flow cytometry. A FLAG tag was included to facilitate the detection of isoform 2. HEK cells transfected with CD30L isoforms, with or without a C-terminal FLAG tag (Iso1-FLAG, Iso2-FLAG), robustly expressed CD30L (Figure S1A,B) on the cell surface, as measured by flow cytometry. Additionally, both isoforms could be detected in supernatants from HEK cells transfected with CD30L (Figure S2A), indicating that there is likely some proteolytic shedding of the extracellular domains of both isoforms.

**Figure 3:**
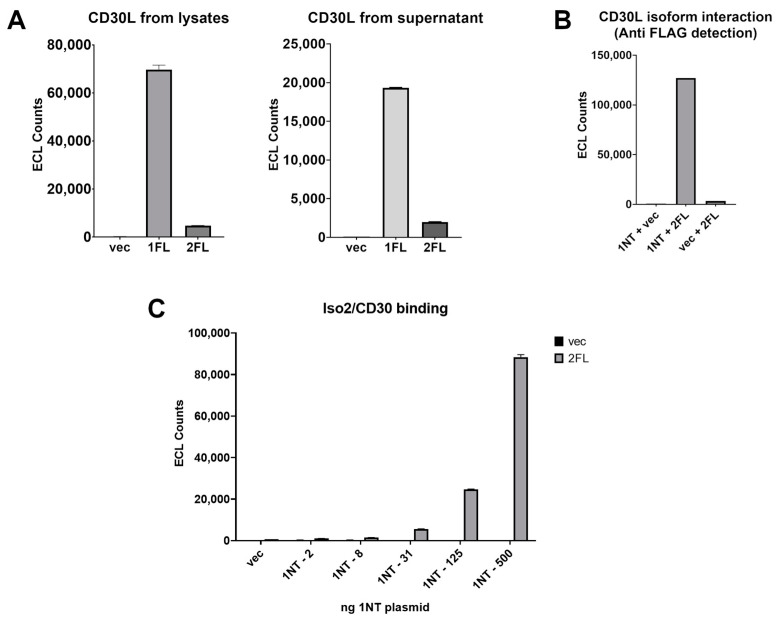
Iso2 has a significantly impaired ability to bind CD30 compared to Iso1, but both are present in an exogenous CD30 protein complex. (**A**) HEK cells were transfected with vector (vec) control or FLAG-tagged Iso1 (1FL) or Iso2 (2FL). Lysates and supernatants were subject to a plate-based binding assay with CD30 coated on the plates and anti-FLAG antibody was used for detection. (**B**) Untagged Iso1 (1NT) was transfected into HEK cells with or without FLAG-tagged Iso2 (2FL), as well as 2FL alone. Cell lysates were subject to a plate-based binding assay. An anti-Iso1-specific CD30L antibody was used for capture and an anti-FLAG antibody for detection. (**C**) HEK cells were transfected with increasing amounts of 1NT plasmid, with either vector (vec, undetectable) control or 2FL at a constant amount and lysates were subject to a plate-based binding assay with recombinant CD30 coated on the plates. Anti-FLAG antibody was used for the detection of 2FL. Error bars for all are from standard deviations between technical replicates; representative experiment of ≥3 shown.

Given that TNF ligand family members are known to form multimeric complexes, we wanted to assess the interactions between CD30 and the CD30L isoforms. To examine this, an antibody sandwich plate-based assay format was developed. We first established that Iso1-FLAG could bind to CD30-coated wells, while CD30 binding for Iso2-FLAG was significantly impaired (Figure 3A). We then wanted to assess whether Iso1 and Iso2 could interact. Wells were coated with an Iso1-specific antibody (Figure S1A) and subject to lysates expressing Iso1 with or without Iso2-FLAG, or Iso2-FLAG alone. Anti-FLAG antibody detected Iso2-FLAG only when Iso1 was expressed, indicating that Iso1 is capable of binding Iso2-FLAG directly or via a protein complex (Figure 3B). From this result, we hypothesized that the CD30L isoforms might both be present in a complex with CD30. Using CD30 recombinant protein-coated wells with lysates from HEK cells transfected with increasing quantities of Iso1 together with vector control or Iso2-FLAG, we observed an Iso1 dose-dependent binding of Iso2-FLAG to an Iso1/CD30 complex (Figure 3C). These data suggest that Iso1 must be present for Iso2 to form a complex with CD30. This tripartite complex was also observed with CD30L isoforms that shed into the supernatant (Figure S2B). 

### 3.4. Iso1 but Not Iso2 Induces Inflammatory Signaling in CD30+ Cell Lines

Three cell lines reported to either express CD30 or be responsive to CD30L were grown and analyzed for CD30 and CD30L expression by flow cytometry. Karpas 299 (K299) cells were originally established from a high-grade non-Hodgkin’s lymphoma and had some T cell properties, including the expression of CD4 and CD5 [24]. HDLM-2 and KM-H2 cells are from Hodgkin’s lymphoma, but HDLM-2 are likely of T cell origin while KM-H2 exhibit features of both dendritic and B cells [25,26]. The cells were subject to analysis by flow cytometry and found to all express CD30 as compared to isotype control (Figure S3). The three cell lines exhibited none or a negligible amount of CD30L Iso1 (Figure S3), which is the only isoform that can be detected by flow cytometry using the anti-CD30L antibodies available for testing.

**Figure 4:**
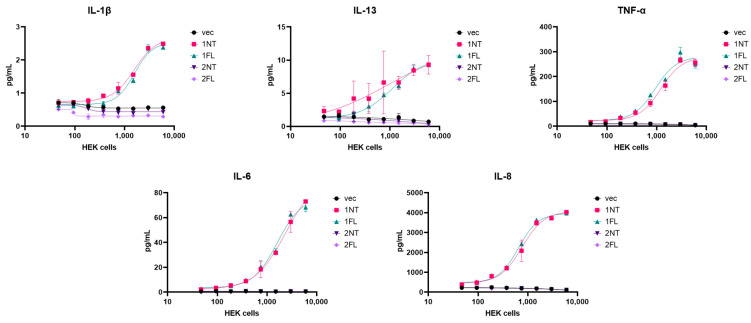
Iso1 induces cytokine production in co-culture with CD30+ cells while Iso2 has no effect. Increasing quantities of HEK cells were transfected with either vector (vec) or CD30L isoforms with (FL) or without (NT) FLAG tag. After transfection, K299 cells were co-cultured with HEK cells for 24 h. Supernatants were harvested and subject to cytokine analysis by MSD assay. Error bars for all are from standard deviations between technical replicates.

To test the functionality of the CD30L isoforms against CD30+ cell lines, we employed a co-culture system. In brief, a monolayer of HEK cells, seeded in increasing number, were transfected with vector control or CD30L isoforms. CD30+ cell lines were then seeded on top for stimulation. K299 were the most efficient at responding to the transfected HEKs (Figure 4), while HDLM-2 and KM-H2 responses to Iso1 were much less robust and fewer cytokines were induced by stimulation. Iso1, but not Iso2, was capable of inducing cytokine production. Cytokine production from K299 cells is shown in Figure 4: IL-1β, IL-13, IL-6, IL-8, and TNF-α were stimulated by Iso1 expression in the co-culture system (Figure 4), while IFN-y, IL-10, IL-4, IL-2, and IL-12-p70 were either not induced or below the limit of detection. The presence of the C-terminal FLAG tag on Iso1 did not hinder its ability to stimulate CD30+ cells (Figure 4).

To determine if a more proximal signaling assay or improved assay sensitivity would reveal any inflammatory signaling capacity of Iso2, we created NFkB luciferase (NFkB-Luc) lines from K299 cells using NFkB-Luc-puro lentivirus. K299 NFkB-Luc-pooled clones responded to recombinant CD30L-Fc protein (Figure S4A) and several K299 NFkB-Luc clones were isolated that had a 10-fold response to CD30L-Fc (Figure S4B), one of which was used in all subsequent K299 NFkB-Luc experiments. Iso1 could stimulate both reporter activity and IL-6 production in the K299 NFkB-Luc clone, while Iso2 could not (Figure S4C,D).

### 3.5. CD30L Iso2 Partially Blocks Iso1-Mediated Signaling

**Figure 5:**
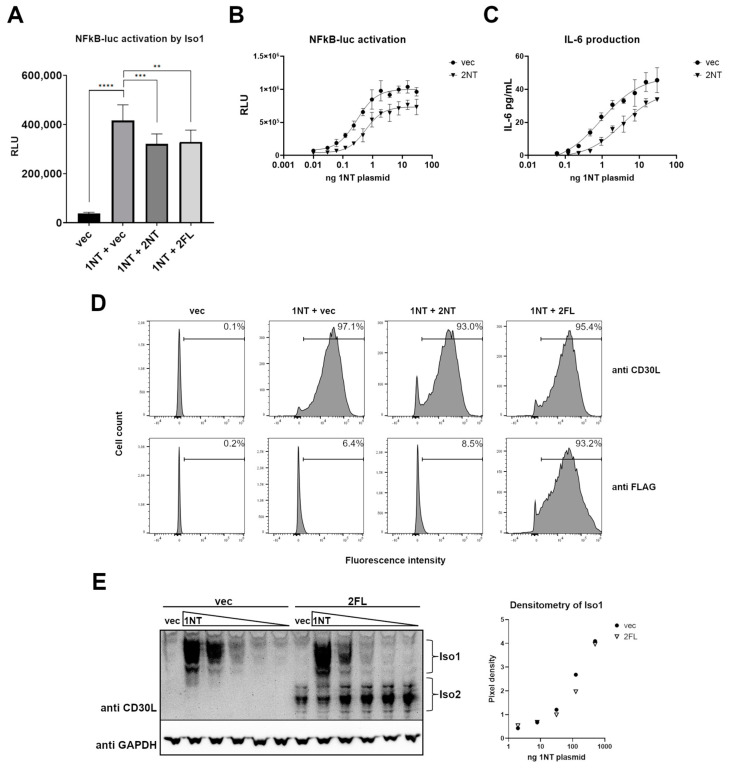
Iso2 partially blocks Iso1-mediated stimulation of K299 cells. (**A**) HEK cells were transfected with either vector (vec) control or untagged Iso1 (1NT) with or without untagged Iso2 (2NT) or FLAG-tagged Iso2 (2FL). Transfected HEK cells were co-cultured with a K299-NFkB-Luc reporter clonal line. Two asterisks indicate *p* ≤ 0.01, three indicate *p* ≤ 0.001, and four indicate *p* ≤ 0.0001. (**B**,**C**) HEK cells were transfected with increasing quantities of 1NT plasmid with either vec or 2NT at a constant quantity, and then co-cultured with the K299-NFkB-Luc reporter line. Reporter activity (**B**) and IL-6 production (**C**) were measured. (**D**) HEK cells were transfected with either 1NT and vec or Iso2 and assessed for 1NT expression by flow cytometry. (**E**) Cells were transfected with increasing quantities of 1NT and either vec or 2FL, and the lysates were subject to SDS-PAGE and Western blot using a pan-CD30L antibody. Densitometry was used to quantify Iso1 bands, normalizing to GAPDH. Error bars for all are from standard deviations between technical replicates; representative experiment of ≥3 shown.

Given the observed complex formation between Iso1 and Iso2 and the lack of ability of Iso2 to signal through CD30, we hypothesized that the presence of Iso2 would prevent optimal Iso1 signaling. To test this, we employed the co-culture model using CD30L isoform-transfected HEK and K299 NFkB-Luc cells. When HEK cells were transfected with Iso1, K299 NFkB-Luc cells had robust reporter activation (Figure 5A). However, when either Iso2 or Iso2-FLAG were co-expressed with Iso1 at equivalent plasmid amounts, there was a 20% reduction in reporter activation (Figure 5A). This decrease in reporter activation from co-transfection of Iso2 was similar across a dose range of Iso1, indicating that Iso2 could not fully abrogate the effects of Iso1 at any transfection ratio (Figure 5B). Supernatants from Figure 5B also showed the reduction in Iso1-mediated signaling as measured by IL-6 production (Figure 5C). This difference in signaling was not due to changes in the percent of cells expressing Iso1 in the presence of Iso2 as measured by flow cytometry (Figure 5D). Additionally, no significant change in the total amount of Iso1 protein expressed was observed by Western blot when HEK cells were transfected with increasing amounts of Iso1 plasmid with either Iso2 or vector control at constant amounts (Figure 5E).

### 3.6. CD30L Iso2 Blocks Iso1 Interaction with CD30

**Figure 6:**
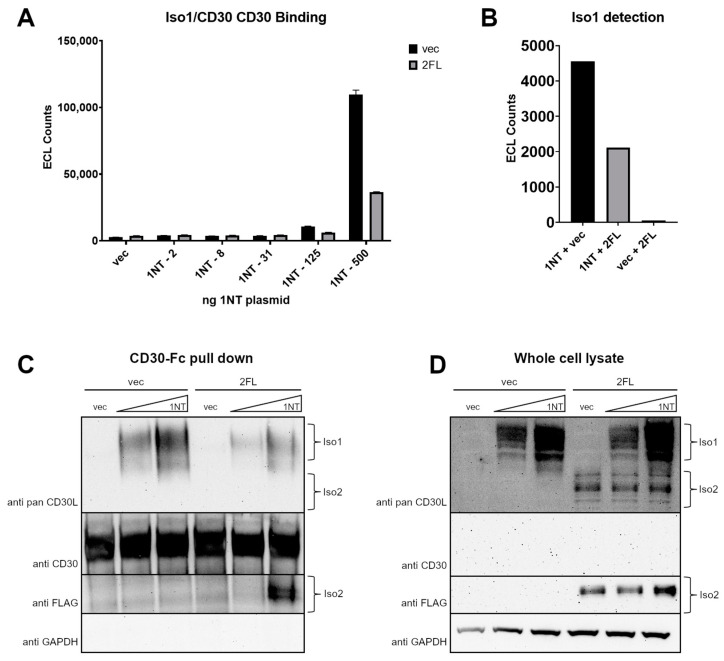
Iso2 can block Iso1/CD30 interaction and anti-Iso1 antibody binding. (**A**) HEK cells were transfected with increasing quantities of untagged Iso1 (1NT) plasmid with a constant amount of either vector (vec) control or FLAG-tagged Iso2 (2FL). Lysates were used in a plate-based binding assay using CD30 recombinant protein as the capture and an Iso1-specific anti-CD30L antibody was used for detection. (**B**) HEK cells were transfected with 1NT with or without 2FL, and 2FL alone. The lysates were used in a plate-based binding assay using an anti-pan-CD30L antibody for capture and an Iso1-specific anti-CD30L antibody for detection. (**C**,**D**) HEK cells were transfected with two quantities of 1NT and a constant quantity of 2FL or vector (vec) control in 10 cm plates. Additionally, 10% of the lysates were set aside as the input whole-cell lysate fraction (**C**), and the remainder was incubated with CD30-Fc recombinant protein and protein A/G beads (**D**). Both were subject to SDS-PAGE and Western blot. Error bars for all are from standard deviations between technical replicates; representative experiment of ≥3 shown.

One explanation for the effects of Iso2 on Iso1 signaling in K299 NFkB-Luc was that Iso2 disrupts Iso1/CD30 interaction. We tested this hypothesis with a plate-based binding assay using wells coated with CD30 and lysates from HEK cells co-transfected with CD30L isoforms, utilizing an Iso1-specific anti-CD30L antibody for detection. HEK cells were transfected with increasing amounts of Iso1 and a constant amount of Iso2 or vector control. When Iso2 was co-transfected, the amount of Iso1 detected in this assay was significantly reduced (Figure 6A). We hypothesized that this could be a consequence of two possibilities. One, the Iso1 antibody epitope was obscured by Iso2, or, two, that Iso2 interaction with Iso1 blocked the ability of Iso1 to bind CD30. To address the possibility of Iso2 directly blocking anti-Iso1 antibody binding, we employed a sandwich plate-based assay where an anti-pan-CD30L antibody was used for capture, followed by lysates from isoform co-transfected cell lysates. The presence of Iso2-FLAG blocked the recognition of Iso1 by the Iso1-specific antibody (Figure 6B).

However, the fact that Iso2 may block the epitope of Iso1-specific antibody recognition did not preclude the possibility that Iso2 may also block the binding of Iso1 to CD30. To test the potential interference of Iso2 in Iso1 binding CD30, we employed a pull-down followed by SDS-PAGE to remove any possibility of epitope blocking before antibody recognition. HEK cells were transfected with vector control or Iso1 at varying quantities, with additional Iso2-FLAG or vector control. Then, 10% of the lysate from these transfected cells were set aside for SDS-PAGE as the input fraction. The remainders of the lysates were incubated with CD30-Fc and protein A/G beads to bind the Fc region. When the pull-down fractions were analyzed with a pan-anti-CD30L antibody, the samples co-expressing Iso2-FLAG had a significantly lower quantity of Iso1 that was pulled-down compared to the vector control (Figure 6C), despite there being no changes in Iso1 levels in the input lysates (Figure 6D). The pan-anti-CD30L was not sensitive enough to pick up co-precipitated FLAG-tagged Iso2 (Figure 6C) as it could in the input lysates (Figure 6D), with anti-FLAG antibody being sensitive enough to detect Iso2 in both. Together, these results indicate that while Iso2 does not alter the level of Iso1 protein when co-expressed, it can block the capacity of Iso1 to bind CD30.

## 4. Discussion

The findings presented here describe isoform 2 of the CD30 ligand for the first time. While both isoforms of CD30L can be found present in a complex with CD30, Iso1 is the only one capable of pro-inflammatory signaling. Not only does Iso2 have no apparent role in pro-inflammatory signaling, but it can also restrict the ability of Iso1 to stimulate NFkB activation and cytokine production. We hypothesized that this negative regulation stems from the ability of the CD30L isoforms to interact. Indeed, we were able to show that in a pull-down assay, the presence of Iso2 in an Iso1/CD30 complex can displace Iso1, perhaps replacing a pure oligomer of Iso1 with hetero-oligomers of both isoforms, thereby reducing the signaling capacity of the ligand/receptor complex.

Given the above, we conclude that Iso1 is the major isoform to be targeted for the therapeutic treatment of IBD. Our findings also suggest that Iso2 may play a role as a potential negative regulator of the CD30 signaling pathway and should be considered in the development of a drug targeting CD30L Iso1. It is possible that Iso2 may mask epitopes which CD30 or anti-CD30L Iso1 antibodies may recognize. Consideration of the CD30L hetero-oligomer is recommended for understanding the biological impact of the therapeutics targeting CD30L that are currently in development. It would be important to choose CD30L Iso1-targeting antibodies that are not inhibited by the presence of Iso2.

The predicted structure of the CD30L extracellular domain that is shared between the isoforms includes an alpha helix. Alpha helices can be structures which mediate protein–protein interactions (PPIs), and this helix is a likely candidate for the hetero-association of the CD30L isoforms. The interaction between CD30L isoforms is at least membrane-associated. However, because both CD30L isoforms undergo proteolytic shedding of their extracellular domains, it is possible that soluble hetero-oligomers of CD30L exist, with evidence of a soluble protein complex consisting of at least Iso1 and Iso2-FLAG binding recombinant CD30 in a plate-based assay (Figure S2B, right panel). The shed complex adds an additional nuance to the function of CD30L and questions remain about the signaling potency of shed hetero-oligomers.

The limitations of the current work include the dearth of Iso2-specific antibodies, especially one that would be reactive in flow cytometry. Such a reagent would allow for a deeper understanding of CD30L isoform expression on the surface of various PBMC cell types and the presence and stoichiometry of endogenous, shed hetero-oligomers in blood. An additional limitation to this study was the difficulty in generating recombinant soluble CD30L protein. It was possible to generate small quantities of Iso1, but the biological activity of these were extremely limited compared to commercial sources of CD30L Iso1 recombinant protein. We were not able to generate any Iso2 protein at all. Algorithmic predictions of protein structure (such as in Figure 1B) show that the extracellular domain of Iso2 is completely disordered in comparison to Iso1, which presents challenges for the expression, solubility, and purification of such a protein. Recombinant protein would have allowed us to test if soluble CD30L Iso2 could be used to reduce Iso1-mediated inflammatory signaling. If such a reduction could be demonstrated, an Iso2 protein could form the basis of a novel biologic drug to target the CD30 signaling pathway in IBD and other inflammatory diseases.

This study on the existence and function of CD30L Iso2 invites a more general question regarding protein isoform function, which has only been previously explored at the gene and proteomic levels. Isoforms have been found for 72% of human genes. These genes express splice variants that encode proteins with different sequences, including secreted proteins, of which two-thirds have isoforms with different subcellular localizations [27]. The increase in complexity for the study of disease etiology and drug development cannot be understated. As canonical forms of gene products tend to be studied, their more obscure isoforms may be ignored despite potentially having regulatory functions, or completely independent functions, when compared to the canonical transcript of the genes and the pathway to which they contribute.

## Data Availability

All relevant data are contained within the manuscript.

## Supplementary Materials

The following supporting information can be downloaded at: https://www.mdpi.com/article/10.3390/cimb46030172/s1, Figure S1: CD30L isoforms with or without FLAG tag can be expressed in HEK cells via transient transfection. HEK cells were transfected for 48 h with either untagged CD30L Iso1 (1NT) or Iso2 (2NT) or FLAG tagged CD30L Iso1 (1FL) or Iso2 (2FL). The cells were subject to flow cytometry using (A) an Iso1 specific antibody (FAB1028) and (B) anti FLAG antibody. Representative experiment of ≥3 shown; Figure S2: Transfected HEK cells shed soluble CD30L isoforms which can bind CD30. (A) HEK cells growing in serum free media were transfected with FLAG tagged Iso1 (1FL) or Iso2 (FL) or vector (vec) control. The supernatants were clarified and subject to a FLAG antibody sandwich plate-based assay. Rabbit anti FLAG was used for capture and mouse anti FLAG was used for detection. (B) HEK cells were transfected with untagged Iso1 (1NT) with or without 2FL. The supernatants were subject to a plate-based binding assay with CD30-Fc recombinant protein coated on the wells. In the left panel, the detection antibody was an Iso1 specific anti CD30L antibody. In the right panel, an anti FLAG tag antibody was used. Error bars for all are from standard deviation between technical replicates, representative experiment of ≥3 shown; Figure S3: Expression of CD30 and CD30L in CD30+ cell lines. Flow cytometry was conducted on three CD30+ cell lines: K299, HDLM-2, and KM-H2. All three showed CD30 expression and no expression of CD30L (Iso1 specific antibody). Red peak is specific antibody signal, blue peak is fluorescently labeled isotype control. Percentage reflects the total cells stained by specific antibody (CD30L or CD30) as gated using isotype control; Figure S4: Characterization of CD30+ cell lines after stable transduction of NFkB-Luc reporter. K299 cells were transduced with an NFkB-Luc lentivirus. (A) Pooled clones were derived from selection and subject to stimulation with increasing quantities of CD30L-Fc recombinant protein, which induced NFkB reporter activation in K299-NFkB-Luc. (B) Clonal selection was successfully done for K299 and several clones responded robustly to CD30L-Fc with NFkB reporter activation, including Clone 7 which was used in the remaining NFkB reporter experiments. (C) HEK cells were seeded at increasing quantity and transfected with either Iso1 or Iso2, with or without FLAG tag (1NT, 2NT, 1FL, 2FL). Transfected HEK were then used to stimulate Clone 7 K299-NFkB-Luc in co-culture. Iso1 induced both (C) NFkB reporter activity and (D) IL-6 production in Clone 7. Error bars for all are from standard deviation between technical replicates, representative experiment of ≥3 shown.

## References

[1] Smith C.A., Gruss H.J., Davis T., Farrah T., Baker E., Sutherland G.R., Brannan C.I., Copeland N.G., Jenkins N.A., Armitage R.J. (1993). CD30 antigen, a marker for Hodgkin’s lymphoma, is a receptor whose ligand defines an emerging family of cytokines with homology to TNF. Cell.

[2] van der Weyden C.A., Pileri S.A., Feldman A.L., Whisstock J., Prince H.M. (2017). Understanding CD30 biology and therapeutic targeting: A historical perspective providing insight into future directions. Blood Cancer J..

[3] Gruss H.J., Boiani N., Williams D.E., Armitage R.J., Smith C.A., Goodwin R.G. (1994). Pleiotropic effects of the CD30 ligand on CD30-expressing cells and lymphoma cell lines. Blood.

[4] Powell I.F., Li T., Jäck H.M., Ellis T.M. (1998). Construction and expression of a soluble form of human CD30 ligand with functional activity. J. Leukoc. Biol..

[5] Wright C.W., Rumble J.M., Duckett C.S. (2007). CD30 activates both the canonical and alternative NF-κB pathways in anaplastic large cell lymphoma cells. J. Biol. Chem..

[6] Nagata S., Ise T., Onda M., Nakamura K., Ho M., Raubitschek A., Pastan I.H. (2005). Cell membrane-specific epitopes on CD30: Potentially superior targets for immunotherapy. Proc. Natl. Acad. Sci. USA.

[7] Savani M., Oluwole O., Dholaria B. (2021). New targets for CAR T therapy in hematologic malignancies. Best Pract. Res. Clin. Haematol..

[8] Othman T., Herrera A., Mei M. (2021). Emerging therapies in relapsed and refractory hodgkin lymphoma: What comes next after brentuximab vedotin and pd-1 inhibition?. Curr. Hematol. Malig. Rep..

[9] Tinazzi E., Barbieri A., Rigo A., Patuzzo G., Beri R., Gerli R., Argentino G., Puccetti A., Lunardi C. (2014). In rheumatoid arthritis soluble CD30 ligand is present at high levels and induces apoptosis of CD30(+)T cells. Immunol. Lett..

[10] Gracias D.T., Sethi G.S., Mehta A.K., Miki H., Gupta R.K., Yagita H., Croft M. (2021). Combination blockade of OX40L and CD30L inhibits allergen-driven memory TH2 cell reactivity and lung inflammation. J. Allergy Clin. Immunol..

[11] Giacomelli R., Passacantando A., Parzanese I., Vernia P., Klidara N., Cucinelli F., Lattanzio R., Santori E., Cipriani P., Caprilli R. (1998). Serum levels of soluble CD30 are increased in ulcerative colitis (Uc) but not in Crohn’s disease (Cd). Clin. Exp. Immunol..

[12] Somada S., Muta H., Nakamura K., Sun X., Honda K., Ihara E., Akiho H., Takayanagi R., Yoshikai Y., Podack E.R. (2012). CD30 ligand/CD30 interaction is involved in pathogenesis of inflammatory bowel disease. Dig. Dis. Sci..

[13] Mei C., Wang X., Meng F., Zhang X., Gan L., Wang Y., Sun X. (2021). CD30L+ classical monocytes play a pro-inflammatory role in the development of ulcerative colitis in patients. Mol. Immunol..

[14] Mei C., Meng F., Wang X., Yan S., Zheng Q., Zhang X., Fu W., Xue J., Wang S., He Y. (2022). CD30L is involved in the regulation of the inflammatory response through inducing homing and differentiation of monocytes via CCL2/CCR2 axis and NF-κB pathway in mice with colitis. Int. Immunopharmacol..

[15] Flores C., Francesconi C.F., Meurer L. (2015). Quantitative assessment of CD30+ lymphocytes and eosinophils for the histopathological differential diagnosis of inflammatory bowel disease. J. Crohn’s Colitis.

[16] Liu J.Z., van Sommeren S., Huang H., Ng S.C., Alberts R., Takahashi A., Ripke S., Lee J.C., Jostins L., Shah T. (2015). Association analyses identify 38 susceptibility loci for inflammatory bowel disease and highlight shared genetic risk across populations. Nat. Genet..

[17] Hong S.N., Park C., Park S.J., Lee C.K., Ye B.D., Kim Y.S., Lee S., Chae J., Kim J.-I., Kim Y.-H. (2016). Deep resequencing of 131 Crohn’s disease associated genes in pooled DNA confirmed three reported variants and identified eight novel variants. Gut.

[18] Huang H., Fang M., Jostins L., Mirkov M.U., Boucher G., Anderson C.A., Andersen V., Cleynen I., Cortes A., Crins F. (2017). Fine-mapping inflammatory bowel disease loci to single-variant resolution. Nature.

[19] Sun X., Yamada H., Shibata K., Muta H., Tani K., Podack E.R., Iwakura Y., Yoshikai Y. (2010). CD30 ligand is a target for a novel biological therapy against colitis associated with Th17 responses. J. Immunol..

[20] Sun X., Somada S., Shibata K., Muta H., Yamada H., Yoshihara H., Honda K., Nakamura K., Takayanagi R., Tani K. (2008). A critical role of CD30 ligand/CD30 in controlling inflammatory bowel diseases in mice. Gastroenterology.

[21] Madeira F., Pearce M., Tivey A.R.N., Basutkar P., Lee J., Edbali O., Madhusoodanan N., Kolesnikov A., Lopez R. (2022). Search and sequence analysis tools services from EMBL-EBI in 2022. Nucleic Acids Res..

[22] Kelley L.A., Mezulis S., Yates C.M., Wass M.N., Sternberg M.J.E. (2015). The Phyre2 web portal for protein modeling, prediction and analysis. Nat. Protoc..

[23] Longo P.A., Kavran J.M., Kim M.S., Leahy D.J. (2013). Transient mammalian cell transfection with polyethylenimine (Pei). Methods Enzymol..

[24] Fischer P., Nacheva E., Mason D.Y., Sherrington P.D., Hoyle C., Hayhoe F.G., Karpas A. (1988). A Ki-1 (Cd30)-positive human cell line (Karpas 299) established from a high-grade non-Hodgkin’s lymphoma, showing a 2;5 translocation and rearrangement of the T-cell receptor beta-chain gene. Blood.

[25] Drexler H.G., Gignac S.M., Hoffbrand A.V., Leber B.F., Norton J., Lok M.S., Minowada J. (1989). Characterization of Hodgkin’s disease derived cell line HDLM-2. Recent Results Cancer Res..

[26] Uehira K., Amakawa R., Ito T., Uehira T., Ozaki Y., Shimizu T., Fujimoto M., Inaba M., Fukuhara S. (2001). A Hodgkin’s disease cell line, KM-H2, shows biphenotypic features of dendritic cells and B cells. Int. J. Hematol..

[27] Uhlén M., Fagerberg L., Hallström B.M., Lindskog C., Oksvold P., Mardinoglu A., Sivertsson Å., Kampf C., Sjöstedt E., Asplund A. (2015). Proteomics. Tissue-based map of the human proteome. Science.

