# Supplementary material for: The Opposite Functions of CD30 Ligand Isoforms
Source: Curr Issues Mol Biol. 2024 Mar 21;46(3):2741–56. doi: 10.3390/cimb46030172 (PMC10969620; doi:10.3390/cimb46030172)
Supplement: Supplementary file 1 [file cimb-46-00172-s001.zip › cimb-2909175-supplementary.pdf]

Supplementary Figures:

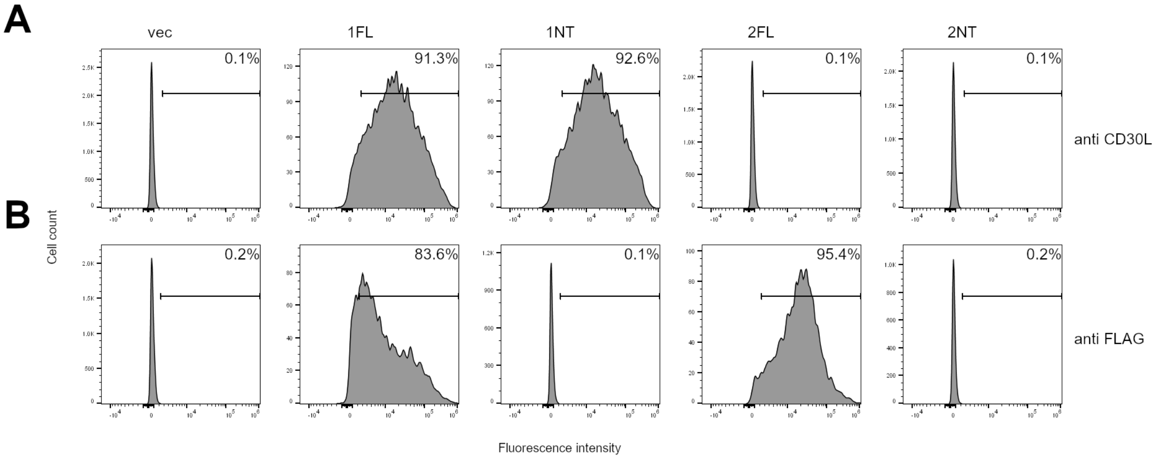

Figure S1. CD30L isoforms with or without FLAG tag can be expressed in HEK cells via transient transfection. HEK cells were transfected for 48 hours with either untagged CD30L Iso1 (1NT) or Iso2 (2NT) or FLAG tagged CD30L Iso1 (1FL) or Iso2 (2FL). The cells were subject to flow cytometry using (A) an Iso1 specific antibody (FAB1028) and (B) anti FLAG antibody. Representative experiment of ⩾ 3 shown.

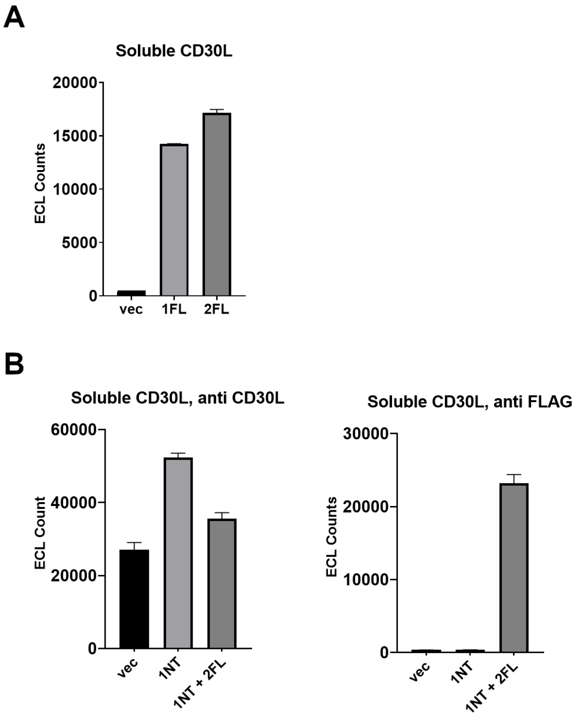

Figure S2. Transfected HEK cells shed soluble CD30L isoforms which can bind CD30. (A) HEK cells growing in serum free media were transfected with FLAG tagged Iso1 (1FL) or Iso2 (FL) or vector (vec) control. The supernatants were clarified and subject to a FLAG antibody sandwich plate-based assay. Rabbit anti FLAG was used for capture and mouse anti FLAG was used for detection. (B) HEK cells were transfected with untagged Iso1 (1NT) with or without 2FL. The supernatants were subject to a plate-based binding assay with CD30-Fc recombinant protein coated on the wells. In the left panel, the detection antibody was an Iso1 specific anti CD30L antibody. In the right panel, an anti

FLAG tag antibody was used. Error bars for all are from standard deviation between technical replicates, representative experiment of ⩾3 shown.

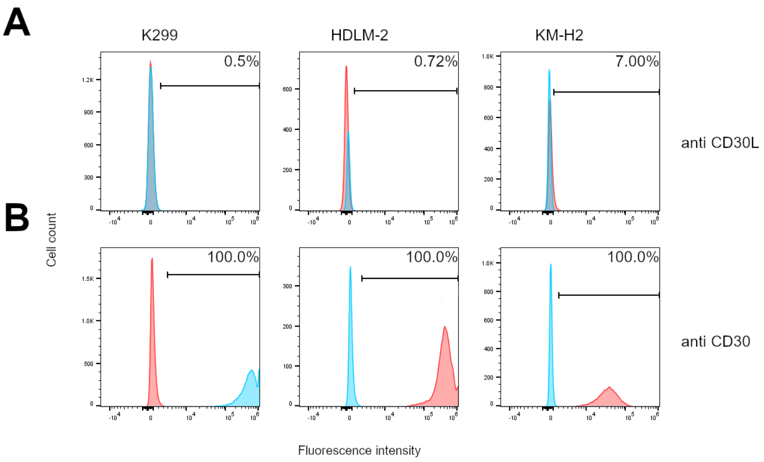

Figure S3. Expression of CD30 and CD30L in CD30+ cell lines. Flow cytometry was conducted on three CD30+ cell lines: K299, HDLM-2, and KM-H2. All three showed CD30 expression and no expression of CD30L (Iso1 specific antibody). Red peak is specific antibody signal, blue peak is fluorescently labeled isotype control. Percentage reflects the total cells stained by specific antibody (CD30L or CD30) as gated using isotype control.

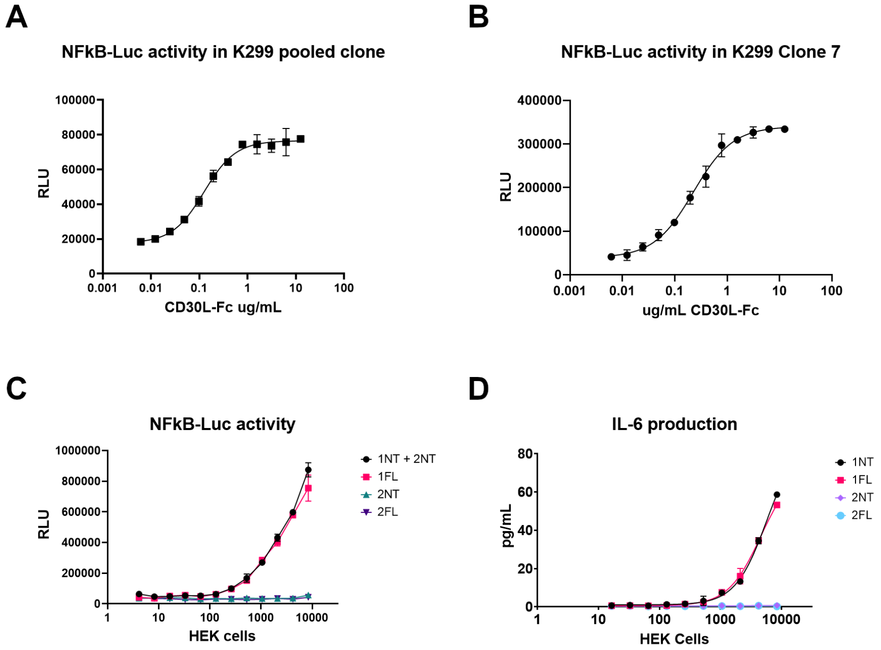

Figure S4. Characterization of CD30+ cell lines after stable transduction of NFkB-Luc reporter. K299 cells were transduced with an NFkB-Luc lentivirus. (A) Pooled clones were derived from selection and subject to stimulation with increasing quantities of CD30L-Fc recombinant protein, which induced NFkB reporter activation in K299-NFkB-Luc. (B) Clonal selection was successfully done for K299 and several clones responded robustly to CD30L-Fc with NFkB reporter activation, including Clone 7 which was used in the remaining NFkB reporter experiments. (C) HEK cells were seeded at increasing quantity and transfected with either Iso1 or Iso2, with or without FLAG tag (1NT, 2NT, 1FL, 2FL). Transfected HEK were then used to stimulate Clone 7 K299-NFkB-Luc in co-culture. Iso1 induced both (C) NFkB reporter activity and (D) IL-6 production in Clone 7. Error bars for all are from standard deviation between technical replicates, representative experiment of ⩾3 shown.
